# Genetic Dissection of Epistatic Interactions Contributing Grain Yield Variability in Rice under Drought

**DOI:** 10.2174/1386207324666210713112127

**Published:** 2021-12-30

**Authors:** Ratna Rani Majumder, Nitika Sandhu, Shailesh Yadav, Margaret Catolos, Ma. Teresa Sta. Cruz, Paul Cornelio Maturan, Lutful Hassan, Mohammad Amir Hossain, Arvind Kumar

**Affiliations:** 1 Rice Breeding Platform, International Rice Research Institute, DAPO Box 7777, Metro Manila, Philippines;; 2 Bangladesh Agricultural University, Mymensingh 2202, Dhaka, Bangladesh;; 3 Punjab Agricultural University, Ludhiana, Punjab 141004, India;; 4 IRRI South Asia Regional Centre (ISARC), NSRTC Campus, Varanasi-221006, Uttar Pradesh, India

**Keywords:** Drought tolerance, epistatic interaction, pyramided lines (PLs), QTL, rice, grain yield

## Abstract

**Aims:**

The aim of the present study was to evaluate the performance of ‘high’-‘low’ yielding pyramided lines (PLs), having the same combinations of *qDTYs* in Samba Mahsuri, MR219 and IR64-Sub1 genetic backgrounds, and to understand the genetic interactions among QTL and/with genetic background affecting grain yield.

**Background:**

Epistasis regulates the expression of traits governed by several major/minor genes/QTL. Multiple pyramided lines (PLs) with the same grain yield QTL (*qDTYs*) combinations but possessing grain yield variability under different levels of reproductive stage drought stress were identified in different rice genetic backgrounds at International Rice Research Institute (IRRI).

**Objectives:**

The objectives of the present study were to evaluate the performance pyramided lines (PLs) with drought QTL in the backgrounds of Samba Mahsuri, MR219 and IR64*-*Sub1 under reproductive stage drought stress (RS) and NS (non-stress) conditions, to understand the effect of epistatic interactions among *qDTYs* and with genetic background on GY under the differential level of stress and to identify the promising drought-tolerant lines with high yield under drought and higher background recovery in different genetic backgrounds.

**Methods:**

The experiments were conducted in 2015 DS (dry season), 2015 WS (wet season) and 2017 DS at IRRI, Los Baños, Philippines, in a transplanted lowland ecosystem under lowland severe stress (LSS), lowland moderate stress (LMS) and lowland non-stress (LNS). The experiments were laid out in alpha lattice design with two replications.

**Results:**

Several digenic interactions were found in different genetic backgrounds, 13 interactions in Samba Mahsuri, 11 in MR219 and 20 in IR64-Sub1 backgrounds. Among all digenic interactions, one QTL × QTL interaction, 17 QTL × background and 26 background × background interactions resulted in GY reduction in low yielding PLs in different genetic backgrounds under LSS or LMS. Negative interaction of *qDTY_3.1_*, *qDTY_4.1_* and *qDTY_9.1_* with background markers and background × background interactions caused up to 15% GY reduction compared to the high yielding PLs under LMS in the Samba Mahsuri PLs. In MR219 PLs, the negative interaction of *qDTY_2.2_*, *qDTY_3.2_*, *qDTY_4.1_* and *qDTY_12.1_* with the background marker interval RM314-RM539, RM273-RM349 and RM445-RM346, RM473D-RM16, respectively resulted 52% GY reduction compared to the high yielding PLs under LSS. In IR64-Sub1 PLs, *qDTY_6.1_* interacted with background loci at RM16-RM135, RM228-RM333, RM202-RM287 and RM415-RM558A marker interval under LSS and at RM475-RM525 marker interval under LMS, causing GY reduction to 58% compared to the high yielding PLs.

**Conclusion:**

High yielding PLs in Samba Mahsuri (IR 99734:1-33-69-1-22-6), MR219 (IR 99784-156-87-2-4-1) and IR64-Sub1 (IR 102784:2-89-632-2-1-2) backgrounds without any negative interactions were identified. The identified selected promising PLs may be used as potential drought-tolerant donors or may be released as varieties for drought-prone ecosystems in different countries.

## 1 INTRODUCTION

Global climate change is affecting the agriculture, health, socio-economic, environmental status in the world as the sea level is rising and the heat waves becoming more intense. Drought stress is considered a major constraint to rice production in rainfed lowlands, which cover almost 30% of the world’s total rice area, threatening the livelihood of many poor farmers along with their families. In this situation, smart and potential technologies are a crucial need for the existing unstable climatic situation. A rice variety, which is high yielding and stress-tolerant could be the best option to cope with the existing climate change.

Understanding the role of epistatic interactions on the phenotypic expression of complex traits is challenging. Epistatic interactions need to be considered in exploiting the differential expression of genotypes harboring genomic regions for different abiotic/biotic stress resistance/tolerance traits in different genetic backgrounds. Recent advances in molecular markers and genomics technologies have provided new opportunities to explore such unknown interactions. Epistatic non-allelic interactions are one of the important genetic factors making a substantial contribution to the variation in complex traits [1], such as grain yield. Epistasis can either exert additive or dominance effect. In some cases, it can completely mask the effect of a gene, or it can modify the effects of a gene, unmasking the effect of a gene that remains dormant [2]. Epistasis interactions between different pairs of alleles may influence a single character [2]. Genetic backgrounds can also play a significant role in improving the positive effect of QTL/genes for complex traits [3, 4].

GY under drought is a complex trait and has been reported to be controlled by various major and minor QTL. Drought breeding programs at IRRI had successfully identified different major QTL (*qDTY_1.1_, qDTY_2.1_, qDTY_2.2_, qDTY_3.1_, qDTY_3.2_, qDTY_4.1,_ qDTY_6.1_* and *qDTY_12.1_*) with a large and consistent effect on GY under drought [5]. The potential drought-tolerant line can produce a good yield under stress conditions as well as no yield reduction under non-stress conditions. In recent years, successful efforts have been made to introgress and pyramid the stable QTL for GY under reproductive stage drought in mega rice varieties; IR64, Swarna, Samba Mahsuri, TDK1, MRQ74 and MR219 using marker-assisted breeding strategy [6 -8]. Pyramiding of QTL using marker-assisted selection (MAS) is a feasible breeding strategy to maximize the phenotypic level of a complex trait [9, 10]. Moreover, different QTLs could perform differently in the different genetic backgrounds, resulting in differential expression of introgressed QTL in different genetic backgrounds. The pyramiding of QTLs in developing rice varieties with multiple tolerance is a better option with respect to plant breeders. Several examples of QTL/genes pyramiding into elite germplasms through MAS techniques improved tolerance to several biotic and abiotic stresses in many crops, including rice, wheat, cotton, *etc.* [11, 12].

The examples include the development of promising lines involving pyramiding of bacterial blight resistance genes *Xa4, xa5, xa13* and *Xa21* into CRMAS2621-7-1 (Improved Lalat) [13]. The successful introgression of the *Sub1* gene was reported in IR64 to develop submergence-tolerant rice varieties by Septiningsih *et al.* [14]. The pyramiding of *qCTF7, qCTF8* and *qCTF12* showed increased cold tolerance at the fertilization stage [10].

However, the introgression of multiple QTL/genes in different genetic backgrounds does not always result in the improvement of the targeted trait(s). The phenotyping expression of the complex traits, such as grain yield under drought in such pyramided lines, may be lower or higher than the expected level due to various interactions [6, 15-18]. Therefore, these interactions must be avoided/exploited for the successful deployment of QTL pyramiding through the MAS approach. The appropriate number of molecular markers are required to estimate the background recovery for the better coverage of the genome coverage as it may be helpful in eliminating the linkage drag due to the non-random association of alleles or linkage disequilibrium associated with different introgressed loci. Furthermore, the desirable allele introgressed in one particular genetic background may become undesirable in another background due to the negative interactions with other introgressed loci and with genetic background.

Genomic interactions (QTL × QTL, QTL × background and background × background interaction) reported to play an important role in the drought MAS breeding program, significantly affecting the expression of introgressed *qDTY*s [2]. In this context, a number of QTL × QTL interactions have been reported in MAS PLs carrying different *qDTY* in various genetic backgrounds [6, 7, 10, 19]. Most of these studies identified additive QTL × QTL interaction and reported yield increase under drought stress without any yield reduction under non-stress or control conditions. The effect of epistatic interactions of QTL with background markers had been identified recently for various complex traits in several crop species, including rice [20-22]. However, there are few reports revealing all sorts of possible interactions like QTL × QTL, QTL × background and background × background interaction existing in low-yielding PLs as compared with high-yielding PLs, having the same QTL combination and same genetic background. However, in other crops like wheat, maize, soybean and cassava, the GY reduction has been reported due to the epistatic interactions [23-26]. Keeping this in view, the present study has been undertaken, i) to evaluate the performance PLs with drought QTL in the backgrounds of Samba Mahsuri, MR219 and IR64*-*Sub1 under reproductive stage drought stress (RS) and NS (non-stress) conditions, ii) to understand the effect of epistatic interactions among *qDTYs* and with genetic background on GY under different level of stress (LNS, LMS and LSS), and iii) to identify the promising drought-tolerant lines with high yield under drought and higher background recovery in different genetic backgrounds.

## 2 MATERIALS AND METHODS

### 2.1 Plant Materials

The experiments were conducted in the experimental field of IRRI, Los Baños, Laguna, Philippines (14°30`N longitude 121^0^15`E latitude). After evaluation of several pyramided lines in different genetic backgrounds across different seasons, successive generation advancement (F_2_ to F_7_/F_8_ generation advance), and different conditions (LNS, LMS and LSS), seven pairs of PLs with the same *qDTYs* combination in the background of Samba Mahsuri, MR219 and IR64- Sub1 but possessing differential yield were selected for further study (Table **1**). All other PLs generated through MAS under different backgrounds were presented in Table **S1**. The PLs in the Samba Mahsuri background were developed through marker-assisted backcrossing of Samba Mahsuri with IR 87728-75-B-B, donor of *qDTY_2.2_* and *qDTY_4.1_* [6]. MR219 PLs were generated through marker-assisted backcrossing of MR219 with three donors IR 77298-14-1-2-10 (*qDTY_2.2_* donor), IR 81896-B-B-195 (*qDTY_3.1_* donor) and IR 84984-83-15-18-B (*qDTY_12.1_* donor) [8]. IR64-Sub1 PLs were developed through the intercrossing of IR64-Sub1 with IR 74371-46-1-1 and Way Rarem carrying *qDTY_12.1_* (*qDTY_12.1_* donor) [5]. The PLs used in the present study were re-confirmed for the presence of different *qDTYs* with earlier reported markers (Table **S2**).

### 2.2 Evaluation of the PLs Possessing *qDTYs* under LMS, LSS and LNS Conditions

The experiments were conducted in dry seasons (DS) and wet seasons (WS) of 2015 and DS of 2017 in a transplanted lowland ecosystem under LMS, LSS drought and LNS conditions at the experimental station of IRRI, Los Baños, Philippines. The experiments were laid out in alpha lattice design with two replications. Across seasons, the PLs were screened along with drought-resistant and susceptible checks in the two-rows plot of 5 m in length, maintaining the spacing of 20 cm × 20 cm. Twenty-one days old seedlings were transplanted in the main field. Recommended doses of nitrogen:phosphorus:potassium (N:P:K) was applied at the rate of 120:30:30 kg ha^-1^. Potassium and phosphorus were applied as basal doses. Nitrogen was applied in three equal splits at 10, 30 and 45 days after transplanting (DAT). In the reproductive stage drought stress trial, two doses of N fertilizer were applied at 10 and 30 DAT before initiating the drought stress and a third dose was applied along with life-saving irrigation. The long-duration PLs were planted 15-20 days earlier than the early maturing PLs. In the control non-stress experiments, three to four irrigations were applied as and when necessary till the crop maturity. In stress trial, irrigation was maintained until 30 DAT and excess water was drained out to initiate reproductive stage drought stress until the crop maturity. Perch water table depth was measured at regular intervals by PVC pipe, which were installed in soil up to 1 m depth and 15 cm above the soil surface in stress experiments. Life-saving irrigation was provided at a level of severe stress when all the susceptible checks started showing severe leaf rolling with minimum probability to recover upon watering and perch water table maintained below 1 m. Life-saving irrigation that was provided through flash flooding was drained out after 24 hours to start a new cycle of stress.

### 2.3 Phenotypic Observations

Data on plant height (PH, in cm), days to 50% flowering (DTF, in days) and grain yield (GY, in kg ha^-1^) in all the control and drought stress experiments was recorded. DTF was measured from the date of sowing to the date when 50% of the rice plants in the plot showed flowering. The data on PH was collected from the soil surface to the tip of the plants from three random plants. Plants were harvested at 80% maturity, threshed, seeds were oven-dried to 14% moisture and plot yield was measured and converted to kg ha^-1^ [27].

### 2.4 Genotyping of PLs

The genotyping studies were carried out at genotyping service laboratory (GSL), IRRI, Philippines. Fresh and young leaves of 14 days old, transplanted seedlings of PLs were collected with their respective recipients and donors. Genomic DNA was extracted using a modified Murray and Thompson [28] CTAB protocol, dissolved in 200 μl of TE (Tris-EDTA) buffer and stored at -20°C. PCR amplification was carried out using a thermal cycler (G-Storm GS1, UK). The total reaction mixture of 15 μl constituted of genomic DNA (10 ng), PCR buffer (1×), dNTPs (100 μM), oligonucleotide primers (100 μM) and Taq polymerase (1 unit). The PCR reaction products were then resolved using high-resolution 6-8% (v/v) polyacrylamide gel electrophoresis (PAGE) (CBS scientific, model MGV-202-33) and running in a TBE buffer (1×) at 90 volts for 1.5 - 2 hrs, depending on the product size of the SSR microsatellite markers. After electrophoresis, separation of the DNA fragments staining with SYBER Safe™ and visualization under UV trans-illuminator (AlphaImager™ System) was performed. A total of ~600 microsatellite markers equally distributed on all the 12 rice chromosomes were selected from the rice database, Gramene (http://www.gramene.org/), and further used to study the polymorphism among the recipient parents and their respective PLs. The PLs in Samba Mahsuri (*qDTY_2.2_+qDTY_4.1_*), MR219 (*qDTY_2.2_+qDTY_3.1_+qDTY_12.1_*) and IR64-Sub1 (*qDTY_12.1_+Sub1*) backgrounds were first genotyped using the foreground markers earlier reported by Bernier *et al.* [29], Dixit *et al.* [30], Venuprasad *et al.* [27], Vikram *et al.* [31] and Swamy *et al.* [8] to confirm the presence of reported QTL. The PLs included in the study were thereafter genotyped using background-specific markers (Samba Mahsuri, MR219, IR64-Sub1) evenly distributed over all chromosomes. For background genotyping, a total of 80, 102 and 120 polymorphic SSR markers in Samba Mahsuri, MR219 and IR64-Sub1 backgrounds, respectively, were chosen. The polymorphic background-specific markers distributed 20 cM apart from each other in the genome were selected for each background and used further to calculate the background genome recovery.

### 2.5 Statistical Analysis

The phenotypic data obtained from all the experiments were analyzed for calculating the trial means, standard error of difference (SED) and heritability (H) using the statistical IRRI Plant Breeding Tools (PB Tools v1.4, [32] software. The least significant difference (LSD) at *p* = 0.05 significance was used to compare the means of the PLs and infer the significant differences of the traits studied between parents and each PLs. ANOVA (analysis of variance) was calculated as follows using a linear mixed model.


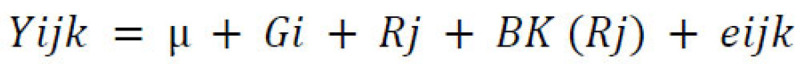


Where, the measurement recorded in each plot represented as Yijk, overall mean as µ, the effect of i^th^ genotype as Gi, the effect of the j^th^ replicate as Rj, block effect of j^th^ replicate as BK (Rj) and the error as eijk. While estimating entry means, the effect of PLs was considered as fixed, and the replication and block effects were considered as random.

### 2.6 Background Recovery

The background recovery (BC) percentage of the selected PLs in each background was estimated with the following formula, earlier used by Sundaram *et al.* [33].


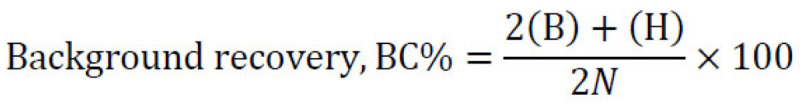


Where, SSR marker loci which are homozygous for the genetic background are represented as ‘B’, the number of marker loci that are in heterozygous state represented as ‘H’, the total number of polymorphic SSR markers used for background estimation are represented as ‘N’.

### 2.7 Epistatic Interaction Analysis

Graphical Genotype (GGT v 2.0) software was used for the graphical representation of the background genome [34]. QTL IciMapping ver. 4.0.1. software was used to estimate the epistatic interactions between the microsatellite markers loci [35]. The two-stage stepwise regression analysis was used to detect the most significant microsatellite markers and the microsatellite markers pair followed by 2D scanning (two-dimensional) to detect the significant digenic interactions using the phenotypic values estimated based on the best fitted multiple regression model [36]. The threshold LOD (logarithm of odds) value was calculated using a permutation test that involves 1000 runs at *p* = 0.01 to detect the significant digenic interactions between microsatellite marker loci. While mapping, the window size and walk speed for the genome scan kept as 10 cM and 1 cM, respectively. The epistatic interactions were classified negative or positive based on reduction or enhancement of GY.

## 3 RESULTS

### 3.1 Phenotypic Performance of the PLs Carrying *qDTYs*

The drought stress at the reproductive stage was classified as LMS, LSS and overstressed based on the observed yield reduction compared with the LNS control [37]. The overstressed trials were excluded from the analysis. Across seasons (WS, DS) and years (2015-2017), the PL possessing the same *qDTY*(s) showed contrasting GY under similar levels of drought stress. Based on GY performance, the PLs were classified into high yielding and low yielding PLs (Tables **1** and **2**). The mean yield reduction of PLs with *qDTY_2.2_+qDTY_4.1_* QTL combination in Samba Mahsuri background ranged from 94.1 to 97.2% under LSS and 78.1 to 85.0% under LMS as compared to LNS (Table **2**).

Under LSS, the pyramided line IR 99734:1-33-69- 1-22-6 showed higher GY (307 kg ha^-1^) followed by IR 99734:1-33-69-1-12-8 (278 kg ha^-1^), however, Samba Mahsuri had the lowest GY (48 kg ha^-1^). Under LMS, the grain yield varied from 970 kg ha^-1^ for IR 99734:1-33-69-1-12-9 to 1135 kg ha^-1^ for IR 99734:1-33-69-1-22-6. In LNS, IR 99734:1-33-69-1-12-10 recorded a yield of 6555 kg ha^-1^, followed by IR 99734:1-33-69-1-12-8 (6525 kg ha^-1^), whereas the yield of Samba Mahsuri was observed as 5735 kg ha^-1^ (Table **3**).

The DTF of the PLs ranged from 79 to 95 days under LSS, 83 to 85 days under LMS and 82 to 84 days under LNS. There was no significant difference observed in DTF among PLs of Samba Mahsuri under LNS. PH of PLs was drastically reduced in LSS as compared to LMS and LNS. Under LMS and LNS, the PLs showed significant differences for PH when compared to the parents. Moderate to high heritability of GY (0.45 to 0.92) was found under LSS, LMS and LNS.

The GY under LSS for the PLs in MR219 background varied from 703 kg ha^-1^ to 1463 kg ha^-1^ with trial mean GY 587 kg ha^-1^ (Table **2**). Under LSS, among the PLs containing *qDTY_2.2_ +qDTY_3.1_+qDTY_12.1,_* IR 99784-255-78-2-3-1 yielded 1463 kg ha^-1^ followed by 789 kg ha^-1^ for IR 99784- 188-202-1-1-1 and 739 kg ha^-1^ for IR 99784-255-7-1-4-1. Among the PLs possessing *qDTY_3.1_+qDTY_12.1_* in MR219 background, IR 99784-156-87-2-4-1 yielded 1085 kg ha^-1^ followed by 724 kg ha^-1^ for IR 99784-255-7-2-6-1 and 703 kg ha^-1^ for IR 99784-255-7-2-4-1. All the PLs produced significantly higher GY as compared to the parent MR219 (44 kg ha^-1^) under LSS conditions. Under LMS, GY of the PLs containing *qDTY_3.1_+ qDTY_12.1_* were higher than the PLs with *qDTY_2.2_+qDTY_3.1_+ qDTY_12.1._* IR 99784-156-87-2-4-1 with *qDTY_3.1_+qDTY_12.1_* showed 1645 kg ha^-1^ GY, while IR 99784-255-7-2-6-1 with the same QTL combination showed 1165 kg ha^-1^. MR219 produced a low yield of 295 kg ha^-1^ under LMS. The grain yield reduction of the PLs ranged from 68.0 to 90.2% in LSS, 64.1 to 82.7% in LMS compared to LNS. Significant differences in DTF were observed among the PLs and parents across seasons. DTF of PLs ranged from 78 to 88 days under LSS, 77 to 85 days under LMS and 77 to 85 days under LNS. Heritability for GY was 0.78, 0.89 and 0.58 under LSS, LMS and LNS, respectively in the MR219 background.

In IR64 Sub1 background, the PLs possessing *qDTY_12.1_+Sub1* produced GY ranged from 413 kg ha^-1^ (IR 102783:2-70-21-1-1-4) to 984 kg ha^-1^ (IR 102784:2-89- 632-2-1-2) with trial mean GY 594.4 kg ha^-1^, while IR64- Sub1 was not harvested under LSS (Table **2**). Under LMS, IR 102783:2-70-1-2-1-1 yielded the highest (1610 kg ha^-1^), while IR 102783:2-70-21-1-1-4 produced poor yield (675 kg ha^-1^). The yield reduction ranged from 82.9 to 93.3% under LSS and 68.2 to 89.0% under LMS compared to the GY under LNS. Heritability for GY was 0.78, 0.89 and 0.58 under LSS, LMS and LNS, respectively. Significant differences in DTF and PH were observed among the PLs and the parent.

### 3.2 Background Recovery of the PLs

Background genome recovery is important for the removal of undesirable genetic parts with the incorporation of desirable homozygous gene/QTL of interest of the introgressed lines or recombinants. A total of 80, 102 and 120 SSR markers were used to calculate the background recovery [36] for the PLs in Samba Mahsuri, MR219 and IR64-Sub1 backgrounds carrying *qDTYs*, respectively (Table **4**). Background recovery of Samba Mahsuri PLs varied from 50 to 68% and the highest was captured in IR 99734:1-33-69-1-12-9 (Table **2**). Similarly, in the MR219 background, the highest background recovery was observed in IR 99784-188-202-1-1-1. Among IR64-Sub1 PLs, background recovery ranged from 80 to 88%, with maximum background recovery in IR 102783:2-70-1-2-1-1. The promising PLs captured higher recurrent genome background and yielded well under LSS, LMS and LNS conditions, presented in Table **3**.

### 3.3 Epistatic Interactions Among *qDTYs* and with Genetic Background

**Fig. (1):**
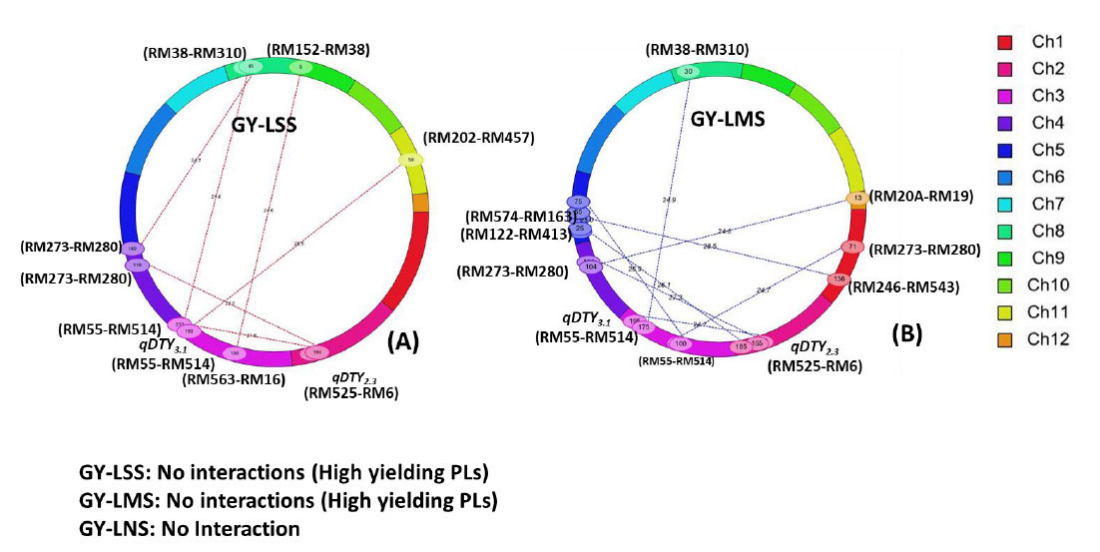
Cyclic illustrations of interactions among the low yielding PLs of Samba Mahsuri background for GY, **A**) Epistasis of GY QTLs under LSS condition, **B**) Epistasis of GY QTLs under LMS condition. (*A higher resolution / colour version of this figure is available in the electronic copy of the article*).

A total of 13 digenic interactions were observed in low-yielding PLs in Samba Mahsuri background under LSS and LMS conditions (Table **S3**). Among the detected 13 interactions, 8 interactions were found between *qDTY* QTL and background marker loci and remaining 5 interactions were detected among the background markers loci located on different chromosomes in the genome. The *qDTY_4.1_* on chromosome 4 had shown negative interaction with the background loci covering the genetic region between the marker loci RM525-RM6 (on chromosome 2) under LSS (Table **S3**, Fig. **1A**); RM6-RM425 (on chromosome 2) and RM20A-RM19 (on chromosome 12) under LMS (Table **S3**, Fig. **1B**). Similarly, the *qDTY_3.1_* located in the marker interval of (RM55-RM514) on chromosome 3 had shown negative interaction with the background markers pair RM38-RM310 (on chromosome 8) and RM202-RM457 on chromosome 11 under LSS condition (Table **S3**, Fig. **1A**). It is worth mentioning that we found some additional QTL × background interactions, such as the marker interval of RM296-RM524 on chromosome 9 (*qDTY_9.1_*), also showed negative interaction with background marker pair RM563-RM16 on chromosome 3 in Samba Mahsuri PLs under LSS condition (Table **S3**, Fig. **1A**), even though the mentioned QTL was not targeted in our introgression program. In the context of the background × background interactions, a total 5 negative epistatic interactions were observed among background markers located on chromosomes 1, 2, 3 and 5 under LMS condition (Table **S3**, Fig. **1B**). Variation explained under LSS ranged from 4.24 to 7.54% and from 4.23 to 8.80% under LMS (Table **S3**).

**Fig. (2):**
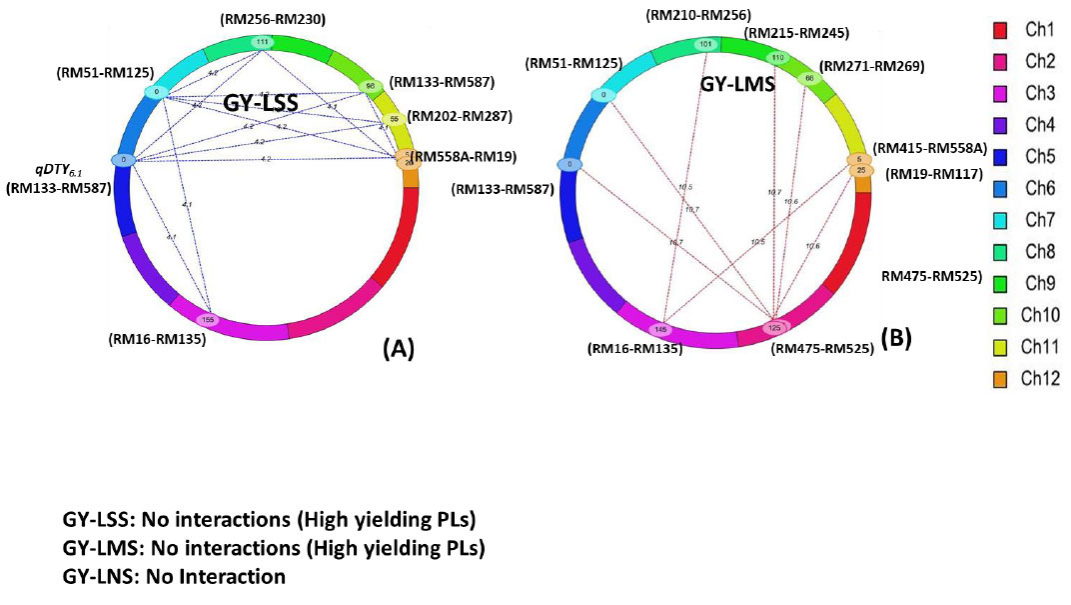
Cyclic illustrations of interactions among the low yielding PLs of MR219 background for GY, **A**) Epistasis of GY QTLs under LSS condition, **B**) Epistasis of GY QTLs under LMS condition. (*A higher resolution / colour version of this figure is available in the electronic copy of the article*).

Among the low yielding MR219 PLs with *qDTY_2.2_+qDTY_3.1_+qDTY_12.1_*, a total of 5 digenic interactions under LSS while 6 interactions under LMS condition (Table **S4**) were observed. Out of the total 11 digenic negative interactions, the identified 4 interactions under LSS were QTL × background markers loci interactions, one was QTL × QTL interaction and the remaining 6 interactions under LMS were observed among background markers loci of the genome. Among the QTL × QTL interactions, *qDTY_3.2,_* located in the marker interval of (RM175-RM36) on chromosome 3, interacted negatively with the *qDTY_4.1_* spanning marker interval between RM273-RM349 on chromosome 4. The *qDTY_2.2_* located in the marker interval of (RM211-RM279) of chromosome 2, interacted negatively with the background marker pair (RM314-RM539) on chromosome 6, while *qDTY_12.1_* located in the marker interval of (RM28048-RM511) on chromosome 12, interacted negatively with the background marker pair (RM473D-RM16) on chromosome 3 and (RM445-RM346) on chromosome 7, as well (Table **S4**, Fig. **2A**). All the other epistatic background × background interactions observed between different marker pairs of chromosomes 1, 2, 3, 8 and 11 (Table **S4**, Fig. **2A** and **2B**) produced a negative effect on GY under LMS conditions.

**Fig. (3):**
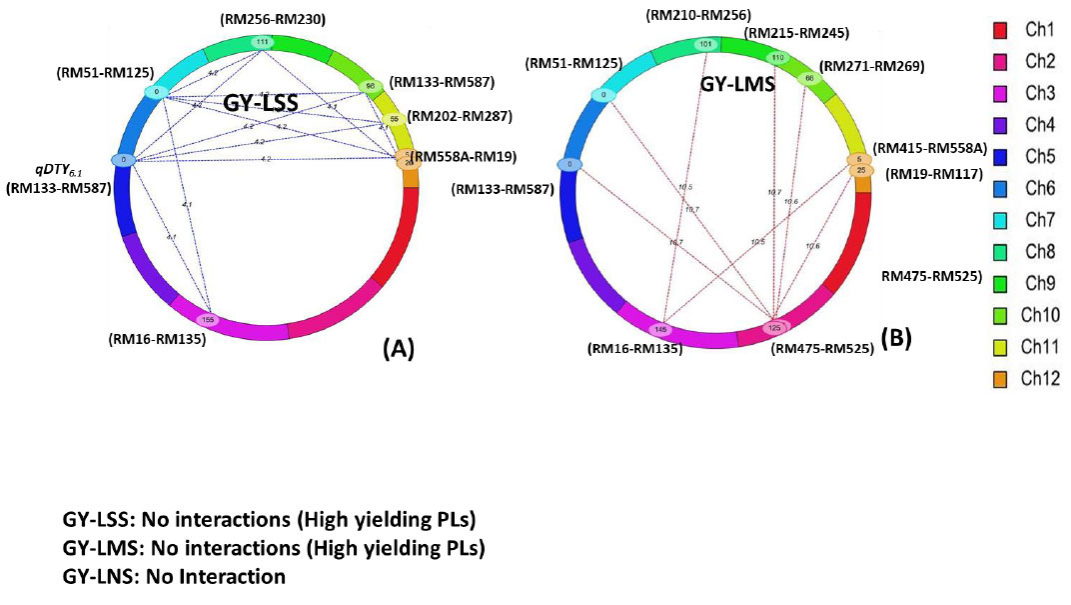
Cyclic illustrations of interactions among the low yielding PLs of IR64-Sub1 background for GY, **A**) Epistasis of GY QTLs under LSS condition, **B**) Epistasis of GY QTLs under LMS condition. (*A higher resolution / colour version of this figure is available in the electronic copy of the article*).

Twenty digenic interactions were found among the low-yielding PLs of IR64-Sub1 background. Among all the interactions, 13 were found in LSS and 7 interactions were found under LMS condition (Table **S5**). A total of 5 digenic interactions were found between *qDTY_6.1_* located in the marker interval of (RM133-RM587) and background marker pairs, and the rest were caused due to background × background marker pair interactions. Under LSS condition, *qDTY_6.1_* interacted negatively with the background marker pairs RM16-RM135 on chromosome 3, RM228-RM333 on chromosome 10, RM202-RM287 on chromosome 11 and with RM415-RM558A on chromosome 12 (Table **S5**, Fig. **3A**). Under the LMS condition, *qDTY_6.1_* interacted negatively with marker pair (RM475-RM525) on chromosome 2 (Fig. **3B**).

*qDTY_6.1_* was not targeted in the background of the IR64- Sub1, but the genomic region spanning *qDTY_6.1_* showed background interactions with genomic regions on different chromosomes among the low yielding PLs of IR64-Sub1. Several other interactions among background marker pairs were observed on chromosomes 2, 3, 6, 7, 8, 11, 12 and 13 under LSS and LMS conditions (Table **S5**). Variation explained under LSS ranged from 2.09 to 4.84% and under LMS from 2.21 to 8.35% (Table **S5**).

No negative interactions were observed among the high yielding PLs under LSS and LMS conditions in Samba Mahsuri (Fig. **1**), MR219 (Fig. **2**) and IR64-Sub1 (Fig. **3**) backgrounds. Furthermore, no significant negative interactions were reported under LNS conditions in both category (high and Low yielding) PLs in Samba Mahsuri (Fig. **1**), in MR219 (Fig. **2**) and IR64-Sub1 (Fig. **3**) backgrounds.

### 3.4 Performances of Promising PLs in Different Backgrounds

Table **2** illustrated the performances of promising PLs under LSS and LMS compared to their respective recipient parent. The high yielding PL IR 99734:1-33-69-1-22-6, carrying *qDTY_2.2_+qDTY_4.1_* in Samba Mahsuri background, showed grain yield advantage of 259 kg ha^-1^ and 852 kg ha^-1^ over the recipient parent Samba Mahsuri under LSS and LMS, respectively. Among the high yielding PLs of MR219, IR 99784-156-87-2-4-1 carrying *qDTY_3.1_*+*qDTY_12.1_* showed a yield advantage of 1085 kg ha^-1^ and 1350 kg ha^-1^ over MR219 under LSS and LMS, respectively. Among the IR64-Sub1 PLs, the grain yield advantage over IR64-Sub1 ranged from 868 kg ha^-1^ to 984 kg ha^-1^ under LSS and 750 kg ha^-1^ to 940 kg ha^-1^ under LMS conditions. Under LNS, the grain yield performance of PLs was nearly like the elite recipient parent for all three studied backgrounds in the present study.

## 4 DISCUSSION

Samba Mahsuri, MR219 and IR64 are popular and high-yielding mega rice varieties but highly susceptible to drought. Several approaches have been followed in the past to introgress major effect QTL for grain yield under reproductive stage drought stress in the genetic background of popular varieties like Samba Mahsuri [6], MR219 [7] IR64 [38], Savitri [39, 40], TDK1-Sub1 and Swarna [41] to produce drought-tolerant lines in backgrounds of these popular rice varieties. Drought tolerance with single drought QTL may not be effective in capturing the expected level of yield increase under drought in the introgression lines. Pyramiding multiple QTL or several desirable alleles in a single breeding line through the MAS approach may provide an opportunity to achieve the desired level of phenotypic variance and yield increase [42-44]. However, in some cases, the effect of pyramiding two or more QTL has not been as expected [45]. Many interactions, such as QTL × QTL, QTL × background and/or QTL × environment, have been reported to influence the additive effect of introgressed QTL in PLs [6, 16, 46]. In this study, PLs combined with various *qDTYs* were generated through marker-assisted breeding using donors for *qDTY* QTL, such as *qDTY_2.2,_* and *qDTY_4.1_,* in the background of Samba Mahsuri, *qDTY_2.2,_ qDTY_3.1,_* and *qDTY_12.1_* in MR219 and *Sub1* along with *qDTY_12.1_* in IR64 background. The stepwise phenotypic and genotypic selection, in addition to the genotypic selection using the peak marker first followed by recombinant markers associated with the introgressed *qDTYs* ensured cost-effective and successful incorporation of the targeted QTL in PLs of respective backgrounds (Table **S2**).

Even though backcrosses were attempted in the present study, we were able to get polymorphism for only 80, 102 and 120 SSR markers after surveyed with ~600 SSR rice microsatellites makers in Samba Mahsuri, MR219 and IR64- Sub1 backgrounds available in the lab, and only polymorphic markers were used to measure the genome recovery. The limitation of the study was the number of markers used to study the background recovery. More markers need to be designed to study the background recovery critically. We observed breeding lines with high background recovery but with a lower yield, which was probably due to linkage drag. The selected promising lines with good yield had background recovery ranging between 50 to 68% in Samba Mahsuri background, 50 to 78% in MR219 background, and 80 to 88% in IR64-Sub1 background, which is low compared to other previously studied backgrounds [38, 41]. Donor segment can be further reduced by using the recombinant selection markers and generation of whole-genome profiling data through SNP-chips [47] of respective *DTY* QTL. Jena *et al.* [48] also explained low background recovery (82-89.5%) in some of the developed rice near isogenic lines for brown plant hopper resistance. Here, the importance to dissect the QTL × QTL, QTL × background and background × background interactions in these backgrounds using the developed introgressed lines with same QTL combination but possessing differential yield under different level of drought stress was highlighted. The study showed that these kinds of interactions led to the reduction in grain yield of low-yielding introgression lines possessing the same QTL combination and having a higher percentage of recurrent parent genome. The selected promising introgression lines were free from such kind of undesirable linkage.

Genome-wide background genotypic data and foreground markers linked with introgressed *qDTYs* had revealed several significant digenic interactions prevailing among low-yielding PLs in all the three genetic backgrounds used in this study. A total of 14, 17 and 20 digenic interactions were found among the low yielded PLs of Samba Mahsuri, MR219 and IR64-Sub1, respectively. Three types of epistatic interactions, such as interactions among QTL, interactions among the introgressed QTL and the background markers and interactions between the background markers of the genome under LSS and LMS conditions, were observed among the low yielding PLs used in the study. Epistasis often occurs when one gene locus masks or modifies the phenotype of a second gene locus where the effect of one gene (locus) is dependent on the presence of one or more 'modifier genes', *i.e.,* the genetic background/QTL. By masking the effect of a gene, epistasis can create interaction between different pairs of alleles influencing a single character [2]. From the digenic interactions reported in the present study, it was easily understood that epistasis played a significant role in hampering the GY improvement in low-yielding PLs [49].

The role of such interactions in rice also reported in various QTL mapping and marker-assisted drought breeding programs under drought [16]. QTL × QTL positive interaction of *qDTY_12.1_* with *qDTY_2.3_* and *qDTY_3.2_* had been previously identified, which resulted in improving GY under drought [16]. Shamsudin *et al.* [7] also found similar findings in BC_1_F_3_ derived lines, the negative interactions of the introgressed regions among themselves and with the genetic background led to the reduction in GY under drought. A significant epistatic interaction of *qDTY_4.1_* + *qDTY_9.1_* locus with *qDTY_7.1_* has been reported earlier in the PLs of Samba Mahsuri, enhancing the GY under drought [6].

PLs developed with various combinations of *qDTYs* in multiple backgrounds of Samba Mahsuri, MR219 and IR64 showed a significant increase in GY under drought (Table **3**) [6-8], Table **5** represents the developed PLs with different QTL combinations in the background of TDK1, Sabitri, IR64 and Swarna [38, 41]. We understood that a positive interaction prevailed among the introgressed QTL in different backgrounds. The lines are high-yielding, with the possibility of no negative interactions among the QTL.

Apart from positive QTL × QTL interactions, many interactions exist in the genome, which can contribute negatively and make the introgression line unfit for cultivation. In the present study, we have detected negative interactions between drought QTL, where *qDTY_3.2_* interacted negatively with *qDTY_4.1_* in MR219 background under LSS, leading to significant reduction in GY under drought.

In this study, in total, 14 QTL × background interactions were found in low-yielding PLs, which may be due to the existing non-random association of drought QTL alleles and the background markers loci. Similarly, Liberman and Feldman [49] reported that the modifier allele invades when there is tight linkage disequilibrium of genes which causes epistatic interactions. In Samba Mahsuri background, negative interactions were observed between *qDTY_3.1_* with the background marker located on chromosome 8 (RM38-RM310) in both LSS and LMS conditions. Similarly, *qDTY_2.2_*, *qDTY_4.1_* and *qDTY_12.1_* interacted negatively with MR219 background marker pairs located on different chromosomes. Interactions of *qDTY_2.2_*, *qDTY_3.2_* and *qDTY_6.1_* with background loci in different genetic backgrounds in the present study were also reported in the genetic background of IR64 and TDK1-Sub1 [40].

These common negative interactions prevailing in multiple genetic backgrounds suggested the possibility of devising a suitable strategy to avoid these negative interactions in pyramiding the above-mentioned QTL in any of the rice genetic backgrounds. Apart from this, QTL × background interactions, QTL × QTL interactions were also observed in the low yielding PLs in all the backgrounds studied.

The effect of genetic backgrounds loci in the expression of target QTL/gene has been discussed earlier for various complex traits in different crops, including rice [3, 4, 50, 51]. However, most of the earlier studies did not report background markers loci interaction with introgressed QTL and their influence on the overall expression of target traits. In the present study, several strong epistatic interactions between the background markers on different chromosomes were reported. Some earlier studies have highlighted the importance of epistatic interactions contributing more than the main effect of the QTL to the total variation of a complex trait in various crops [52, 53]. Under such conditions, the phenotype of the lines was influenced largely by the background markers of the recipient parents [54]. Colocalization of grain yield QTL with QTL for days to 50% flowering and plant height reported previously, explaining the high heritability of grain yield [55].

The negative interactions involving QTL × QTL as well as the QTL × genetic background interactions observed in some of the PLs clearly indicated the need to modify/filter out the QTL/genetic background alleles showing negative interactions with alternate alleles, leading to the grain yield reduction under drought. Finding such epistatic interactions in the present study will be very useful information in selecting the PLs having maximum potentiality in the phenotypic expression of a complex trait such as drought.

The high-yielding promising pyramiding lines with positive or additive interactions/no negative interactions among the pyramided QTL were identified based on their consistent performance across the different levels of stress and non-stress control conditions. The grain quality of some of the selected entries also showed good potentiality of the PLs (Table **S6**). These PLs may have the potential to be released as promising drought-tolerant varieties to combat drought in the drought-prone regions among South Asian countries. The introduction of new favorable alleles through rapid breeding cycles using these PLs through heterosis breeding might be the possible scenario to boost the rate of gain under drought and non-stress conditions because in genomic selection, these lines can add genetic value predicted from high- density markers positioned throughout the genome.

## CONCLUSION

The QTL × QTL, QTL × background and background × background interactions on same/different rice chromosomes affected the GY of low yielding PLs under LSS and LMS conditions in all the three genetic backgrounds (Samba Mahsuri, MR219 and IR64-Sub1) in the present study. No interactions were observed in LNS conditions among all the low and high-yielding PLs. So far, this is the first report on the causes of reduction under the effect of the introgressed QTL in PLs, resulting in poor performance of the breeding lines in Samba Mahsuri, MR219 and IR64 genetic backgrounds. The findings of the study provide new insight to researchers engaged in marker-assisted introgression of QTL governing complex traits and shall help develop strategies to identify and exclude such negative interactions while initiating introgression programs. PLs, having no negative interactions and performing well under LMS, LNS and LNS conditions, might be selected for successful deployment of QTL pyramided lines or could be evaluated for release as a variety in drought-prone areas in different countries.

## Figures and Tables

**Table 1:** List of pyramided lines (PLs) used for QTL interaction studies.

**Recurrent Parent**	**Donor Parent**	**QTL(s)**	**No. of Low Yielding Lines**	**No. of High Yielding Lines**
Samba Mahsuri	IR 87728-75-B-B	*qDTY_2.2_+qDTY_4.1_*	2	2
MR219	IR 77298-14-1-2-10 IR 81896-B-B-195 IR 84984-83-15-18-B	*qDTY_2.2_+qDTY_3.1_+qDTY_12.1_*	2	1
MR219	IR 81896-B-B-195 IR 84984-83-15-18-B	*qDTY_3.1_+qDTY_12.1_*	2	1
IR64-Sub1	Way Rarem IR 74371-46-1-1	*qDTY_12.1_+Sub1*	2	2

**Table 2:** Yield of different pyramided lines (PLs) from different backgrounds under LSS, LMS drought and LNS conditions.

Pyramided Lines (PLs)	QTL(s)	Grain Yield (kg ha^-1^)			Days to Flowering (Days)			Plant Height (cm)			%YR over LNS		Background		Classifi- cation
		WS2015	DS2017	DS2017	WS2015	DS2017	DS2017	WS2015	DS2017	DS2017					
		LSS	LMS	LNS	LSS	LMS	LNS	LSS	LMS	LNS	LSS	LMS			-
IR 99734:1-33-69-1-12-8	*qDTY_2.2_+qDTY_4.1_*	278	1022	6525	86	83	83	60	60	93	95.7	87.6	Samba Mahsuri		HY
IR 99734:1-33-69-1-22-6	*qDTY_2.2_+qDTY_4.1_*	307	1140	5195	95	85	84	62	62	89	94.1	78.2			HY
IR 99734:1-33-69-1-12-9	*qDTY_2.2_+qDTY_4.1_*	204	970	5210	79	84	82	63	63	98	96.1	81.4			LY
IR 99734:1-33-69-1-12-10	*qDTY_2.2_+qDTY_4.1_*	186	980	6555	83	85	83	66	66	99	97.2	85.0			LY
Samba Mahsuri	*-*	48	288	5735	131	112	96	74	83	83	97.5	90.5	Recipient		-
IR 87728-75-B-B	*qDTY_2.2_, qDTY_4.1_*	-	540	2667	-	85	83	-	94	79			Donor		-
Trial Mean	-	114.7	880	5004	97.9	83	80	70.8	63	94	-	-	-		-
LSD_0.05_	-	182.4	82.3	1371.4	14.32	8.37	5.8	11.6	8.7	9.9	-	-	-		-
Heritability	-	0.45	0.89	0.58	0.86	0.85	0.92	0.61	0.80	0.88	-	-	-		-
Pyramided lines (PLs)	QTL(s)	2015DS	2017DS	2017DS	2015DS	2017DS	2017DS	2015DS	2017DS	2017DS	%YR over LNS		-		-
IR 99784-255-78-2-3-1	*qDTY_2.2_+qDTY_3.1_+ qDTY_12.1_*	1463	1525	7060	80	77	79	65	64	78	68.0	66.7	MR219		HY
IR 99784-156-87-2-4-1	*qDTY_3.1_+qDTY_12.1_*	1085	1645	6530	78	79	77	67	59	89	83.4	74.8	-		HY
IR 99784-255-7-1-4-1	*qDTY_2.2_+qDTY_3.1_+ qDTY_12.1_*	739	1310	7565	86	85	82	69	67	92	90.2	82.7	-		LY
IR 99784-188-202-1-1-1	*qDTY_2.2_+qDTY_3.1_+ qDTY_12.1_*	789	1530	6140	83	80	79	64	67	92	87.1	75.1	-		LY
	-	-	-	-	-	-	-	-	-	-	-	-	-		-
IR 99784-255-7-2-4-1	*qDTY_3.1_+qDTY_12.1_*	703	215	4222	86	-	122	65	-	56	83.3	94.9	-		LY
IR 99784-255-7-2-6-1	*qDTY_3.1_+qDTY_12.1_*	724	1165	3245	88	84	93	60	63	71	77.7	64.1	-		LY
MR219	-	44	295	7408	107	109	97	63	62	99	68.0	66.7	Recipient		-
IR 81896-B-B-195	*qDTY_3.1_* in MR219	-	1720	6290	-	90	104	-	65	119			Donor		-
IR 84984-83-15-18-B	*qDTY_12.1_* in MR219	-	1000	5885	-	79	117	-	56	62			Donor		-
IR 77298-14-1-2-10	*qDTY_2.2_* in MR219	-	1815	5970	-	74	75	-	65	94			Donor		-
Trial Mean	-	587.2	1558	5204	86.0	83	80	61.5	63	94	-	-	-		-
LSD_0.05_	-	435.9	929.1	1371.4	11.8	8.4	5.8	6.1	8.7	9.9	-	-	-		-
Heritability	-	0.77	0.89	0.58	0.88	0.85	0.92	0.17	0.8	0.88	-	-	-		-
IR 102783:2-70-1-2-1-1	*qDTY_12.1_*+*Sub1*	868	1610	5065	76	75	72	59	49	77	82.9	68.2	IR64-Sub1		HY
IR 102784:2-89-632-2-1-2	*qDTY_12.1_*+*Sub1*	984	1420	7285	82	82	78	68	61	89	86.5	80.5	-		HY
IR 102783:2-70-21-1-1-4	*qDTY_12.1_*+*Sub1*	413	675	6130	87	85	81	62	57	97	93.3	89.0	-		LY
IR 102783:2-70-129-2-1-2	*qDTY_12.1_*+*Sub1*	665	710	5840	87	85	83	64	57	94	82.9	87.8	-		LY
IR64-Sub1	-	-	670	6710	105	91	84	47	55	92			Recipient		-
Way Rarem	*qDTY_12.1_* in IR64	191	510	4730	89	96	80	84	80	122			Donor		-
Trial Mean	-	594.4	1558	5450	65.2	83	80	85.0	63	94	-	-	-	-	
LSD_0.05_	-	398	822.0	1371.4	8.4	8.4	5.8	7.3	8.7	9.9	-	-	-	-	
Heritability	-	0.78	0.89	0.58	0.91	0.85	0.92	0.82	0.8	0.88	-	-	-	-	

* DS: Dry Season, LSS: Lowland severe stress, LMS: Lowland moderate stress, LNS: Lowland non-stress, HY: High yielding, LY: Low yielding, YR:Yield reduction, LSD_0.05_: Least Significant difference at the 5% level of significance, Empty cells represents yield could not harvested due to stress.

**Table 3:** Recurrent parent genome recovery of pyramided lines (PLs) from different backgrounds.

Background	Pyramided Lines (PLs)	Parentage	QTL(s)	Recurrent Genome Recovery (%)	Grain Yield (kg ha^-1^)			Classification
					LSS	LMS	LNS	
					WS2015	DS2017	DS2017	
Samba Mahsuri	IR 99734:1-33-69-1-12-8	IR07F287*2/IR 87728-75-B-B	*qDTY_2.2_+qDTY_4.1_*	50	278	1022	6525	HY
Samba Mahsuri	IR 99734:1-33-69-1-22-6	IR07F287*2/IR 87728-75-B-B	*qDTY_2.2_+qDTY_4.1_*	65	307	1140	5195	HY
Samba Mahsuri	IR 99734:1-33-69-1-12-9	IR07F287*2/IR 87728-75-B-B	*qDTY_2.2_+qDTY_4.1_*	68	204	970	5210	LY
Samba Mahsuri	IR 99734:1-33-69-1-12-10	IR07F287*2/IR 87728-75-B-B	*qDTY_2.2_+qDTY_4.1_*	58	186	980	6555	LY
Recipient	Samba Mahsuri	RP 5/Mansur	-	-	48	288	5735	-
Donor	IR 87728-75-B-B	IR 77298-5-6-18/IR 64	-	-	-	540	2667	-
	-	-	-	-	DS2015	DS2017	DS2017	-
MR219	IR 99784-255-78-2-3-1	IR 98003:257/MR 219	*qDTY_2.2_+qDTY_3.1_+qDTY_12.1_*	73	1463	1525	7060	HY
MR219	IR 99784-156-87-2-4-1	IR 98003:257/MR 219	*qDTY_3.1_+qDTY_12.1_*	66	1085	1645	6530	HY
MR219	IR 99784-255-7-1-4-1	IR 98003:257/MR 219	*qDTY_2.2_+qDTY_3.1_+qDTY_12.1_*	73	739	1310	7565	LY
MR219	IR 99784-188-202-1-1-1	IR 98003:257/MR 219	*qDTY_2.2_+qDTY_3.1_+qDTY_12.1_*	75	789	1530	6140	LY
MR219	IR 99784-255-7-2-4-1	IR 98003:257/MR 219	*qDTY_3.1_+qDTY_12.1_*	65	703	215	4222	LY
MR219	IR 99784-255-7-2-6-1	IR 98003:257/MR 219	*qDTY_3.1_+qDTY_12.1_*	50	724	1165	3245	LY
Recipient	MR219	RU 3365-14-1-1/RU 2977-20-2-2	-	-	44	295	7408	-
Donor	IR 81896-B-B-195	IR 55423-01 (NSIC Rc 9)/2*Swarna	-	-	-	1720	6290	-
Donor	IR 84984-83-15-18-B	IR 79971-B-102-B-B/2*Vandana	-	-	-	1000	5885	-
Donor	IR 77298-14-1-2-10	IR 64/Aday Sel//3*IR 64	-	-	-	1815	5970	-
IR64-Sub1	IR 102783:2-70-1-2-1-1	IR 99621-14/IR 99619-361//IRRI 149	*qDTY_12.1_+Sub1*	88	868	1610	5065	HY
IR64-Sub1	IR 102784:2-89-632-2-1-2	IR 99621-181/IR 99620-158//IRRI 149	*qDTY_12.1_+Sub1*	80	984	1420	7285	HY
IR64-Sub1	IR 102783:2-70-21-1-1-4	IR 99621-14/IR 99619-361//IRRI 149	*qDTY_12.1_+Sub1*	84	413	675	6130	LY
IR64-Sub1	IR 102783:2-70-129-2-1-2	IR 99621-14/IR 99619-361//IRRI 149	*qDTY_12.1_+*Sub*1*	83	665	710	5840	LY
Recipient	IR64-Sub1	IR 40931-33-1-3-2/3*IR 64	-	-	-	670	6710	-
Donor	Way Rarem	IR 9669/B 981	-	-	191	510	4730	-

Note: LSS: Lowland severe stress, LMS: Lowland moderate stress, LNS: Lowland non-stress, HY: High yielding, LY: Low yielding, WS: Wet season, DS: Dry season.

**Table 4:** Grain yield of previously reported PLs possessing *qDTY* combinations in the backgrounds of Samba Mahsuri, MR219 and IR64

Samba Mahsuri Background																				
Designation		IR 99734:1-33-69-1-39-6		IR 99734:1-33-69-1-12-8			IR 99734:1-33-304-1-5-8			IR 99734:1-33-69-1-12-9				IR 99734:1-33-304-1-5-10				Samba Mahsuri		Refs.
QTL(s) Season		*qDTY_2.2_ + qDTY_4.1_*		*qDTY_2.2_ + qDTY_4.1_*			*qDTY_2.2_ + qDTY_4.1_*			*qDTY_2.2_ + qDTY_4.1_*				*qDTY_2.2_ + qDTY_4.1_*				-	
^†^DS2013 (^§^RS)		1299		1299			1279			1299				1279				-		[6]
^‡^WS2014 (^§§^NS)		3242		3073			2842			3073				2842				2137		
DS2014 (RS)		624		1109			823			1109				823				0		
DS2016 (RS)		296		272			340			219				250				0		
DS2016 (NS)		5154		4682			4400			4400				4107				4951		
WS2015 (RS)		498		278			175			307				190				48		
WS2015 (NS)		4508		3847			5404			4353				4880				4044		

**MR219 Background**																				
**Designation**	**IR 99784-255-7-2-5**		**IR 99784-255-68-1-5**			**IR 99784-255-7-2-2**			**IR 99784-255-9-1-3**		**IR 99784-156-87-1-9**		**IR 99784-188-179-1-2**			**IR 99784-255-49-1-1**			**MR219**	**Refs.**
QTL(s) Season	*qDTY_3.1_+ qDTY_12.1_*		*qDTY_2.2_+qDTY_3.1_ + qDTY_12.1_*			*qDTY_3.1_+ qDTY_12.1_*			*qDTY_3.1_+ qDTY_12.1_*		*qDTY_2.2_+qDTY_3.1_+ qDTY_12.1_*		*qDTY_3.1_+ qDTY_12.1_*			*qDTY_3.1_+ qDTY_12.1_*			-	
DS2013 (RS)	1346		1611			1346			888		923		1459			2521			13	[8]
DS2013 (NS)	7783		6905			7783			8174		5808		9107			5832			5917	
DS2014 (RS)	1591		1183			983			936		930		927			903			0	
DS2014 (NS)	8383		7782			9438			9014		11672		7419			8152			8322	

**IR64 Background**																				
**Designation**	**IR87707-446-B-B-B**				**IR87707-445-B-B-B**			**IR 87707-182-B-B-B**				**IR87705-72-12-B**			**IR87705-6-8-B**		**IR64**			**Refs.**
QTL(s) Season	*qDTY_2.2_+qDTY_4.1_*				*qDTY_2.2_+qDTY_4.1_*			*qDTY_2.2_+qDTY_4.1_*				*qDTY_2.2_*			*qDTY_4.1_*		*-*			
DS2010 (RS)	2556				2555			1926				1879			2152		636			[9]
DS2010 (NS)	3752				5045			3875				3569			5399		2987			
DS2011 (RS)	3000				3023			2891				1892			2588		1442			
DS2011 (NS)	4388				5844			5225				6090			6208		5435			

Note: †DS: Dry season, ‡WS: Wet season, ^§^RS: Reproductive stage drought, ^§§^NS: Non-stress.

**Table 5:** Grain yield of previously reported PLs possessing *qDTY* combinations in the backgrounds of TDK1, Sabitri, IR64 and Swarna.

TDK1 Background																						
Designation		IR 90266-B-116-1		IR 90266-B-268-1	IR 90266-B-438-1		IR 90266-B-16-1			IR 90266-B-492-1			IR 90266-B-512-1		IR 90266-B-111-1		IR 90266-B-101-1			TDK1		Refs.
QTL(s) Season		*qDTY_6.1_*		*qDTY_3.1_*	*qDTY_3.1_*_+_ *qDTY_6.1_*		*qDTY_3.1_*_+_ *qDTY_6.1_*			*qDTY*_3.1+_ *qDTY*_6.1_			*qDTY_3.1_*_+_ *qDTY_6.1_*		*qDTY_3.1_*_+_ *qDTY_6.1_*		*qDTY_3.1_*_+_ *qDTY*_6.1_		
†DS2011 (^§^SS)		1467		1564	1735		1618			2604			1790		2572		1539			173		[50]
DS2012 (^§§^MS)		2391		1576	1882		2411			2160			2877		2260		2770			2306		
DS2012 (^§§§^NS)		4664		5290	4802		5373			5392			5542		5297		5892			5985		
DS2013 (MS)		1711		2024	1745		1969			1485			2132		2254		2774			896		
DS2013 (NS)		4905		4727	5163		4942			5153			5057		5580		5053			5054		

**Sabitri Background**																						
**Designation**	**IR 106529-20-40-3-1-B**		**IR 106529-20-40-3-2-B**		**IR 106522-35-5-3-1-B**			**IR 106522-41-8-3-B**		**IR 106523-6-35-2-3-B**			**IR 106523-24-35-1-3-B**		**IR 106523-21-28-1-2-B**		**IR 106523-3-9-3-2-B**			**Sabitri**		**Refs.**
QTL(s) Season	*qDTY_3.1_*		*qDTY_3.1_*+ *qDTY_12.1_*		*qDTY_3.1_*+ *qDTY_12.1_*			*qDTY_12.1_*		*qDTY_3.1_*+ *qDTY_12.1_*			*qDTY_3.1_*+ *qDTY_12.1_*		*qDTY_12.1_*		*qDTY_3.1_*					
DS2016 (NS)	5245		5206		6552			4735		5559			3977		5123		5798			4749		[7]
DS2016 (SS)	343		372		546			627		381			893		517		545			0		

**IR64 Background**										
**Designation**	**IR 102793:1-11-66-3-1-1**	**IR 102796-14-65-2-1-1**	**IR 102796-14-124-1-1-3**	**IR 102784:2-42-136-1-1-3**	**IR 102784:2-42-16-1-1-2**	**IR 102784:2-62-481-2-1-1**	**IR 102784:2-118-549-2-1-2**	**IR 102784:2-89-284-1-1-3**	**IR 64**	**Refs.**
QTL(s) Season	*qDTY_1.2_* + *qDTY_12.1_*	*qDTY_1.2_* + *qDTY_12.1_*	*qDTY*_2.2_ + *qDTY_2.3_*	*qDTY*_2.2_ + *qDTY_2.3_*	*qDTY_2.3_* + *qDTY*_3.2_	*qDTY_2.3_* + *qDTY*_3.2_	*qDTY_4.1_* + *qDTY_12.1_*	*qDTY_4.1_* + *qDTY_12.1_*		
DS2015 (SS)	2141	138	996	572	1426	191	852	369	642	[39]
DS2017 (MS)	1455	1050	2235	1255	1860	1255	2125	1600	715	
DS2017 (NS)	4375	5990	5220	4988	5430	5200	6378	6145	5910	

**Swarna Background**										
**Designation**	**IR 96321-1447-651-B-1-1-2**	**IR 96322-34-223-B-1-1-1**	**IR 96322-34-563-B-2-1-1**	**IR 96321-558-257-B-5-1-2**	**IR 94391-131-358-19-B-1-1-1**	**IR 94391-131-358-19-B-6-1-4**	**IR 96322-34-127-B-1-1-**1	**Swarna-Sub1**	**Swarna**	**Refs.**
QTL(s) Season	*qDTY_1.1_* + *qDTY_3.1_* + *Sub1* (93%)	*qDTY_1.1_* + *qDTY_2.1_*+ *qDTY_3.1_* + *Sub1* (94%)	*qDTY_1.1_* + *qDTY_2.1_*+ *qDTY_3.1_* + *Sub1* (90%)	*qDTY_3.1_* + *Sub1* (92%)	*qDTY_3.1_* + *Sub1* (98%)	*qDTY_3.1_* + *Sub1* (92%)	*qDTY_1.1_* + *qDTY_3.1_* +*Sub1* (95%)	*Sub1*		
‡WS2014 (SS)^a^	1155	1105	1100	845	1405	1133	1103	1008	688	[42]
WS2014 (MS) ^a^	2799	3068	2378	2548	3390	3133	2221	2183	1457	
WS2014 (Sub) ^a^	1351	1383	1151	1105	1989	1500	1650	1081	173	
DS2015 (SS)	1525	1566	1758	1679	2307	1870	1349	521	755	
WS2015 (MS)^b^	3796	3589	3542	2017	-	-	-	2658	2739	
WS2016 (SS)^c^	836	1361	1236	-	-	-	-	764	684	

**Note:** †DS: Dry season, ‡WS: Wet season, ^§^SS: Severe drought stress, ^§§^MS: Moderate drought stress, ^§§^NS: Non stress, ^a^Data from Hardinath (Nepal), ^b^Data from Patna (India), ^c^Data from Raipur (India).

## Data Availability

The data sets supporting the results of this article are included within the article.

## LIST OF ABBREVIATIONS

BC: Background Recovery
DAT: Days After Transplanting
DTF: Days to 50% Flowering
GY: Grain Yield
qDTY: Grain yield QTL under drought
HY: High Yielding
LSD: Least Significant Difference (LSD)
LOD: Logarithm of Odds
LMS: Lowland Moderate Stress
LSS: Lowland Severe-Stress
LNS: Lowland Non Stress
LY: Low Yielding (LY)
PVE: Phenotypic Variation Explained
PH: Plant Height
PLs: Pyramided Lines, Percent variation due to epistasis
BR: Background recovery
SSR: Simple Sequence Repeats
SED: Standard Error of Difference

## SUPPLEMENTARY MATERIAL

Table **S1:** Different PLs of IR64 Sub1, Sambha Mahsuri and MR219 generated through pyramiding of different biotic and abiotic genes/QTL. Table **S2:** Linked markers of introgressed QTL used in foreground selection of Samba Mahsuri, MR219 and IR64- Sub1 backgrounds. Table **S3:** Epistatic interaction of major effect loci conferring grain yield under drought of pyramided lines (PLs) from the background of Samba Mahsuri. Table **S4:** Epistatic interaction of major effect loci conferring grain yield under drought of pyramided lines (PLs) from the background of MR219. Table **S5:** Epistatic interaction of major effect loci conferring grain yield under drought of pyramided lines (PLs) from the background of IR64-Sub1. Table **S6:** Grain quality characters of the selected PLs from MR219 and IR64-Sub1 backgrounds. Supplementary material is available on the publisher’s website along with the published article.
