# Supplementary material for: Genetic Dissection of Epistatic Interactions Contributing Grain Yield Variability in Rice under Drought
Source: Curr Genomics. 2021 Dec 30;22(6):468–83. doi: 10.2174/1386207324666210713112127 (PMC8886627; doi:10.2174/1386207324666210713112127)
Supplement: Supplementary file 1 [file CG-22-468_SD1.pdf]

Supplementary Material

Genetic Dissection of Epistatic Interactions Contributing Grain Yield Variability in Rice under Drought

Ratna Rani Majumder[1,2], Nitika Sandhu[1,3], Shailesh Yadav[1], Margaret Catolos[1], Ma. Teresa Sta. Cruz[1], Paul Cornelio Maturan[1], Lutful Hassan[2], Mohammad Amir Hossain[2] and Arvind Kumar[1,4,*]

[1]*Rice Breeding Platform, International Rice Research Institute, DAPO Box 7777, Metro Manila, Philippines;* [2]*Bangladesh Agricultural University, Mymensingh 2202, Dhaka, Bangladesh;* [3]*Punjab Agricultural University, Ludhiana, Punjab 141004, India;* [4]*IRRI South Asia Regional Centre (ISARC), NSRTC Campus, Varanasi-221006, Uttar Pradesh, India*

Table **S1.** Different PLs of IR64 Sub1, Sambha Mahsuri and MR219 generated through pyramiding of different biotic and abiotic genes/QTLs.

**Background**	**Designation**	**2017DS**
IR64 Sub1	IR 102783:2-70-1-1-1-1	$qDTY_{1.2}$+$qDTY_{12.1}$+*Sub1*
IR64 sub1	IR 102783:2-70-1-2-1-1	$qDTY_{12.1}$+*Sub1*
IR64 sub1	IR 102783:2-70-129-2-1-2	$qDTY_{4.1}$+$qDTY_{12.1}$+*Sub1*
IR64 sub1	IR 102783:2-70-21-1-1-4	$qDTY_{12.1}$+*Sub1*
IR64 sub1	IR 102783:2-70-91-2-1-2	$qDTY_{3.2}$+$qDTY_{2.3}$+$qDTY_{2.2}$
IR64 sub1	IR 102784:2-42-121-1-1-2	$qDTY_{12.1}$+$qDTY_{3.2}$+$qDTY_{2.3}$
IR64 sub1	IR 102784:2-42-47-2-1-4	$qDTY_{2.2}$+$qDTY_{4.1}$
IR64 sub1	IR 102784:2-89-632-2-1-2	$qDTY_{12.1}$
IR64 sub1	IR 102796-14-77-2-1-2	$qDTY_{1.2}$+$qDTY_{12.1}$+*Sub1*
Sambha Mahsuri	IR 99734:1-33-69-1-12-10	$qDTY_{2.2}$+$qDTY_{4.1}$
Sambha Mahsuri	IR 99734:1-33-69-1-12-8	$qDTY_{2.2}$+$qDTY_{4.1}$
Sambha Mahsuri	IR 99734:1-33-69-1-12-9	$qDTY_{2.2}$+$qDTY_{4.1}$
Sambha Mahsuri	IR 99734:1-33-69-1-22-6	$qDTY_{2.2}$+$qDTY_{4.1}$
MR219	IR 99784-156-87-2-4-1	$qDTY_{3.1}$+$qDTY_{12.1}$
MR219	IR 99784-188-202-1-1-1	$qDTY_{2.2}$+$qDTY_{3.1}$+$qDTY_{12.1}$
MR219	IR 99784-226-237-1-5-1	$qDTY_{12.1}$
MR219	IR 99784-255-29-1-1-1	$qDTY_{12.1}$
MR219	IR 99784-255-7-1-4-1	$qDTY_{2.2}$+$qDTY_{3.1}$+$qDTY_{12.1}$
MR219	IR 99784-255-7-2-4-1	-
MR219	IR 99784-255-7-2-6-1	$qDTY_{12.1}$
MR219	IR 99784-255-78-2-3-1	$qDTY_{2.2}$+$qDTY_{3.1}$+$qDTY_{12.1}$

1389-2029/21 $65.00+.00**© 2021 Bentham Science Publishers**

Table **S2.** Linked markers of introgresed QTLs used in foreground selection of Samba Mahsuri, MR219 and IR64-Sub1 backgrounds.

Pyramided lines (PLs)	QTLs with marker interval	Background
IR 99734:1-33-69-1-12-8	*DTY*$_{2.2}$ (RM236), *DTY*$_{4.1}$ (RM518)	Samba Mahsuri
IR 99734:1-33-69-1-12-9	*DTY*$_{2.2}$ (RM236), *DTY*$_{4.1}$ (RM518)	Samba Mahsuri
IR 99734:1-33-69-1-22-6	*DTY*$_{2.2}$ (RM236), *DTY*$_{4.1}$ (RM518)	Samba Mahsuri
IR 99734:1-33-69-1-12-10	*DTY*$_{2.2}$ (RM236), *DTY*$_{4.1}$ (RM518)	Samba Mahsuri
IR 99784-255-78-2-3-1	*DTY*$_{2.2}$ (RM154, RM236, RM279, RM12460, OSR17) + *DTY*$_{3.1}$ (RM416, RM16030, RM520) + *DTY*$_{12.1}$ (RM28048, RM511, RM28099, RM28166, CG29430, indel8, RM28130)	MR219
IR 99784-156-87-2-4-1	*DTY*$_{3.1}$ (RM416, RM16030, RM520) + *DTY*$_{12.1}$ (RM28048, RM511, RM28099, RM28166, CG29430, indel8, RM28130)	MR219
IR 99784-255-7-2-4-1	*DTY*$_{3.1}$ (RM416, RM16030, RM520) + *DTY*$_{12.1}$ (RM28048, RM511, RM28099, RM28166, CG29430, indel8, RM28130)	MR219
IR 99784-188-202-1-1-1	*DTY*$_{2.2}$ (RM154, RM236, RM279, RM12460, OSR17) + *DTY*$_{3.1}$ (RM416, RM16030, RM520) + *DTY*$_{12.1}$ (RM28048, RM511, RM28099, RM28166, CG29430, indel8, RM28130)	MR219
IR 99784-255-7-1-4-1	*DTY*$_{2.2}$ (RM154, RM236, RM279, RM12460, OSR17) + *DTY*$_{3.1}$ (RM416, RM16030, RM520) + *DTY*$_{12.1}$ (RM28048, RM511, RM28099, RM28166, CG29430, indel8, RM28130)	MR219
IR 99784-255-7-2-6-1	*DTY*$_{3.1}$ (RM416, RM16030, RM520) + *DTY*$_{12.1}$ (RM28048, RM511, RM28099, RM28166, CG29430, indel8, RM28130)	MR219
IR 102783:2-70-1-2-1-1	*DTY*$_{12.1}$ (RM28048, RM28099, INDEL8, RM28130), *Sub 1* (Art5 + other markers)	IR64-Sub1
IR 102783:2-70-21-1-1-4	*DTY*$_{12.1}$ (RM28048, RM28099, INDEL8, RM28130), *Sub 1* (Art5 + other markers)	IR64-Sub1
IR 102784:2-89-632-2-1-2	*DTY*$_{12.1}$ (RM28048, RM28099, INDEL8, RM28130), *Sub 1* (Art5 + other markers)	IR64-Sub1
IR 102783:2-70-129-2-1-2	*DTY*$_{12.1}$ (RM28048, RM28099, INDEL8, RM28130), *Sub 1* (Art5 + other markers)	IR64-Sub1

Table **S3.** Epistatic interaction of major effect loci conferring grain yield under drought of pyramided lines (PLs) from the background of Samba Mahsuri.

**Trait Name**	**Chromosome 1***	**Position 1[†]**	**Marker interval 1**	**Chromosome 2[‡]**	**Position 2[‡]**	**Marker interval 2**	**LOD[§]**	**PVE (%)[¶]**	**Additive 1[‡‡]**	**Additive 2[‡‡]**	**Additive by Additive[††]**
GY-LSS	2	149.8	RM525-RM6	4	118.5	RM273-RM280 ($qDTY_{4.1}$)	21.98	7.54	6.25	15.25	-15.25
GY-LSS	3	210.0	RM55-RM514 ($qDTY_{3.1}$)	8	35.0	RM38-RM310	21.60	4.24	10.75	10.75	-19.75
GY-LSS	3	100.0	RM563-RM16	9	5.0	RM296-RM524 ($qDTY_{9.1}$)	24.58	4.29	10.75	19.75	-10.75
GY-LSS	3	190.0	RM55-RM514 ($qDTY_{3.1}$)	11	59.0	RM202-RM457	28.53	4.29	6.25	15.25	-15.25
GY-LMS	12	13	RM20A-RM19	4	123.5	RM273-RM280 ($qDTY_{4.1}$)	24.40	5.24	240.00	235.00	-235.00
GY-LMS	1	71.0	RM292-RM140	3	105.0	RM563-RM16	24.70	4.24	232.50	237.50	-237.50
GY-LMS	2	144.8	RM525-RM6	3	195.0	RM55-RM514 ($qDTY_{3.1}$)	24.74	4.23	240.00	235.00	-235.00
GY-LMS	3	175.0	RM55-RM514 ($qDTY_{3.1}$)	8	30.0	RM38-RM310	24.93	4.24	235.00	240.00	-235.00
GY-LMS	5	25.0	RM122-RM413	5	55.0	RM574-RM163	24.99	4.75	232.50	237.50	-237.50
GY-LMS	2	184.8	RM425-RM138	5	20.0	RM122-RM413	26.10	8.80	237.50	232.50	-237.50
GY-LMS	3	100.0	RM563-RM16	5	75.0	RM574-RM163	26.91	4.92	232.50	237.50	-237.50
GY-LMS	2	154.8	RM6-RM425	4	113.5	RM273-RM280 ($qDTY_{4.1}$)	27.33	6.75	240.00	235.00	-235.00
GY-LMS	1	136.0	RM246-RM543	5	45.0	RM574-RM163	28.46	5.10	237.50	232.50	-237.50

[*]Chromosome ID at the first scanning position, [†]Scanning position in cM of the first marker pair, [‡]Chromosome ID at the second scanning position, [‡]Scanning position in cM of the second marker pair, [§]LOD score caused by epistasis effects, [¶]PVE (%): Phenotypic variation explained by epistatic effects, [‡‡]Estimated additive effect of position 1, [‡‡]Estimated additive effect of position 2, [††]Additive by additive epistatic effect at the two scanning positions.

Table **S4.** Epistatic interaction of major effect loci conferring grain yield under drought of pyramided lines (PLs) from the background of MR219.

**Trait Name**	**Chromosome 1***	**Position 1†**	**Marker Interval 1**	**Chromosome 2‡**	**Position 2⁑**	**Marker Interval 2**	**LOD§**	**PVE (%)**	**Additive 1⁑⁑**	**Additive 2‡‡**	**Additive by Additive††**
GY-LSS	3	40.0	RM175-RM36 ($qDTY_{3.2}$)	4	143.5	RM273-RM349 ($qDTY_{4.1}$)	5.28	17.62	195.69	98.53	-318.70
GY-LSS	4	128.5	RM273-RM349 ($qDTY_{4.1}$)	6	35.0	RM314-RM539	7.86	36.34	-77.08	190.56	-424.67
GY-LSS	2	14.8	RM211-RM279 ($qDTY_{2.2}$)	6	45.0	RM314-RM539	4.89	13.60	-106.09	-31.37	-297.74
GY-LSS	3	130.0	RM473D-RM16	12	60.0	RM28048-RM511 ($qDTY_{12.1}$)	6.93	16.08	372.23	46.37	-307.27
GY-LSS	7	40.0	RM445-RM346	12	60.0	RM28048-RM511 ($qDTY_{12.1}$)	6.73	16.09	372.83	47.14	-307.74
GY-LMS	1	31.0	RM428-RM272	2	79.8	RM300-RM341	5.39	5.84	-14.88	122.38	-512.79
GY-LMS	2	29.8	RM279-RM53	2	84.8	RM341-RM475	5.33	5.85	-14.99	122.49	-513.34
GY-LMS	1	51.0	RM576-RM572	3	0.0	RM81B-RM22	4.98	5.68	-1.25	108.75	-495.00
GY-LMS	2	79.8	RM300-RM341	8	79.4	RM25-RM210	5.34	5.85	122.42	-14.93	-513.00
GY-LMS	2	79.8	RM300-RM341	11	45.0	RM4B-RM202	4.99	5.85	122.44	-14.94	-513.15
GY-LMS	3	0.0	RM81B-RM22	11	110.0	RM254-RM144	4.98	5.68	108.75	-1.25	-495.00

*Chromosome ID at the first scanning position, †Scanning position in cM of the first marker pair, ‡Chromosome ID at the second scanning position, ⁑Scanning position in cM of the second marker pair, §LOD score caused by epistasis effects, ¶PVE (%): Phenotypic variation explained by epistatic effects, ⁑⁑Estimated additive effect of position 1, ‡‡Estimated additive effect of position 2, ††Additive by additive epistatic effect at the two scanning position.

Table **S5.** Epistatic interaction of major effect loci conferring grain yield under drought of pyramided lines (PLs) from the background of IR64-Sub1.

Trait Name	Chromosome 1*	Position 1[†]	Marker interval 1	Chromosome 2[‡]	Position 2[⁑]	Marker interval 2	LOD[§]	PVE (%)	Additive 1[⁑⁑]	Additive 2[‡‡]	Additive by Additive[††]
GY-LSS	3	155.0	RM16-RM135	6	0.0	RM133-RM587 ($qDTY_{6.1}$)	4.12	2.46	-8.13	-66.13	-330.38
GY-LSS	3	155.0	RM16-RM135	7	0.0	RM51-RM125	4.12	2.88	-8.13	-66.13	-330.38
GY-LSS	6	0.0	RM133-RM587 ($qDTY_{6.1}$)	8	111.1	RM256-RM230	4.18	2.82	-66.13	-8.13	-330.38
GY-LSS	7	0.0	RM51-RM125	8	111.1	RM256-RM230	4.18	2.09	-66.13	-8.13	-330.38
GY-LSS	6	0.0	RM133-RM587 ($qDTY_{6.1}$)	10	96.3	RM228-RM333	4.18	3.15	-66.13	-8.13	-330.38
GY-LSS	7	0.0	RM51-RM125	10	96.3	RM228-RM333	4.18	2.44	-66.13	-8.13	-330.38
GY-LSS	6	0.0	RM133-RM587 ($qDTY_{6.1}$)	11	55.0	RM202-RM287	4.15	2.85	-66.13	-8.13	-330.38
GY-LSS	7	0.0	RM51-RM125	11	55.0	RM202-RM287	4.15	2.76	-66.13	-8.13	-330.38
GY-LSS	6	0.0	RM133-RM587 ($qDTY_{6.1}$)	12	5.0	RM415-RM558A	4.17	2.56	-66.13	-8.13	-330.38
GY-LSS	7	0.0	RM51-RM125	12	5.0	RM415-RM558A	4.17	2.89	-66.13	-8.13	-330.38
GY-LSS	8	111.1	RM256-RM230	12	20.0	RM558A-RM19	4.11	3.74	-8.13	-66.12	-330.37
GY-LSS	10	96.3	RM228-RM333	12	20.0	RM558A-RM19	4.12	4.84	-8.13	-66.13	-330.38
GY-LSS	12	5.0	RM415-RM558A	12	20.0	RM558A-RM19	4.11	2.16	-8.13	-66.12	-330.38
GY-LMS	2	119.8	RM475-RM525	6	0.0	RM133-RM587 ($qDTY_{6.1}$)	10.72	8.35	-88.13	6.88	-461.88
GY-LMS	2	119.8	RM475-RM525	7	0.0	RM51-RM125	10.72	6.43	-88.13	6.88	-461.88
GY-LMS	3	145.0	RM16-RM135	8	101.1	RM210-RM256	10.51	7.67	6.88	-88.13	-461.88
GY-LMS	2	119.8	RM475-RM525	9	110.0	RM215-RM245	10.68	4.78	-88.13	6.88	-461.88
GY-LMS	2	114.8	RM475-RM525	10	66.3	RM271-RM269	10.65	6.24	-88.13	6.88	-461.88
GY-LMS	3	145.0	RM16-RM135	12	5.0	RM415-RM558A	10.52	2.21	6.88	-88.13	-461.88
GY-LMS	2	124.8	RM475-RM525	12	25.0	RM19-RM117	10.63	3.65	-88.13	6.88	-461.88

[*]Chromosome ID at the first scanning position, [†]Scanning position in cM of the first marker pair, [‡]Chromosome ID at the second scanning position, [⁑]Scanning position in cM of the second marker pair, [§]LOD score caused by epistasis effects, [¶]PVE (%): Phenotypic variation explained by epistatic effects, [⁑⁑]Estimated additive effect of position 1, [‡‡]Estimated additive effect of position 2, [††]Additive by additive epistatic effect at the two scanning position.

1389-2029/21 $65.00+.00© 2021 Bentham Science Publishers

Table **S6.** Grain quality characters of the selected PLs from MR219 and IR64-Sub1 backgrounds.

Back-ground	Designation	To-tal_grains	Chalki-ness	Chalk_0_10	Chalk_10_25	Chalk_25_50	Chalk_50_75	Chalk_gt_75	Aver-age_length	stddev_length	aver-age_width	amylose_content	gelatiniza-tion_temp
**IR64 sub1**	**IR 102783:2-70-1-2-1-1**	**213**	**0.4**	**97.4**	**2.1**	**0.5**	**0.0**	**0.0**	**6.24**	**0.30**	**2.34**	**24.2**	**I**
IR64 sub1	IR 102783:2-70-91-2-1-2	172	2.9	90.9	4.9	2.4	0.6	1.2	6.84	0.37	2.34	18.6	I
IR64 sub1	IR 102784:2-42-121-1-1-2	106	4.4	86.3	6.9	2.9	3.9	0.0	6.62	0.39	2.37	21.5	I
IR64 sub1	IR 102784:2-42-47-2-1-4	124	4.1	78.8	16.9	4.2	0.0	0.0	6.85	0.38	2.40	20.9	I
**IR64 sub1**	**IR 102784:2-89-632-2-1-2**	**164**	**0.6**	**96.8**	**2.6**	**0.6**	**0.0**	**0.0**	**6.62**	**0.29**	**2.30**	**20.9**	**I**
IR64 sub1	IR 102796-14-77-2-1-2	173	0.4	97.4	2.6	0.0	0.0	0.0	6.47	0.29	2.25	21.2	I
**MR219**	**IR 99784-156-87-2-4-1**	**186**	**1.4**	**92.2**	**5.4**	**2.4**	**0.0**	**0.0**	**7.14**	**0.43**	**2.24**	**20.7**	**HI/I**
MR219	IR 99784-226-237-1-5-1	112	9.9	58.4	26.5	10.6	4.4	0.0	6.68	0.52	2.46	20.1	HI/I
MR219	IR 99784-255-29-1-1-1	132	3.5	89.8	5.5	1.6	1.6	1.6	7.01	0.47	2.35	20.5	HI/I
**MR219**	**IR 99784-255-78-2-3-1**	**142**	**2.7**	**89.3**	**5.3**	**4.6**	**0.8**	**0.0**	**7.08**	**0.54**	**2.24**	**21.1**	**HI/I**

*Bold selected entries
